# Ensuring People Living with HIV Inform the Future of HIV Treatment in Low- and Middle-Income Countries: A Scoping Review and Recommendations for a Community-Led Research Agenda

**DOI:** 10.1007/s10461-024-04442-8

**Published:** 2024-07-11

**Authors:** Danielle F. Resar, Rachel Sapire, Benvy Caldwell, Sarah Jenkins, Kenly Sikwese, Jacque Wambui, Brian Nzano, Carolyn Amole

**Affiliations:** 1 Clinton Health Access Initiative, 383 Dorchester Ave, Boston, MA 02127 USA; 2 Afrocab, Lusaka, Zambia

**Keywords:** Long-Acting Antiretrovirals, HIV Treatment, HIV Service Delivery, HIV Product Attributes, End User Preferences

## Abstract

The HIV treatment landscape in low- and middle-income countries (LMICs) is rapidly evolving, exemplified by the expansion of differentiated service delivery (DSD) during the coronavirus disease (COVID-19) pandemic. Long-acting products represent a new frontier that will require a significant redesign of health systems. It is critical to understand service delivery and product preferences of people living with HIV (PLHIV) and ensure evidence generation is guided by community priorities. We conducted a scoping review to identify gaps among preference studies and inform future research. Peer-reviewed articles published from January 2014-May 2022 reporting acceptability or preference data from PLHIV or caregivers for one or more service delivery or product attribute were eligible. Service delivery studies were restricted to LMIC populations while product studies had no geographical restrictions. Based on gaps identified, we consulted advocates to develop community-led research agenda recommendations. Of 6,493 studies identified, 225 studies on service delivery attributes and 47 studies on product preferences were eligible. The most frequently studied delivery models were integration (*n* = 59) and technology-based interventions (*n* = 55). Among product literature, only 15 studies included LMIC populations. Consultation with advocates highlighted the need for research on long-acting products, including among pediatric, pregnant, and breastfeeding PLHIV, PLHIV on second-line regimens, and key populations. Consultation also emphasized the need to understand preferences on clinic visit frequency, side effects, and choice. While the preference literature has expanded, gaps remain around long-acting regimens and their delivery. To fill these gaps, the research agenda must be guided by the priorities of communities of PLHIV.

## Introduction

The landscape of HIV treatment service delivery models and interventions in low- and middle-income countries (LMICs) is rapidly evolving. The World Health Organization’s (WHO) latest guidelines on service delivery for the treatment of people living with HIV (PLHIV) include several new and updated recommendations on the initiation of antiretroviral therapy (ART) outside health facilities, less frequent clinic visits, and integrated service delivery [1]. These updated guidelines are informed by a growing body of evidence on innovative differentiated service delivery (DSD) models, and the recognition that reaching PLHIV with person-centered services that align with their needs and preferences is critical to improving health outcomes [2]. Health system disruptions during the coronavirus disease (COVID-19) pandemic in 2020 also increased the need to expand community-based services and self-care interventions to prevent a reversal of progress made over the past decade of the HIV response. Successful adaptations during the COVID-19 pandemic to ensure continuity of care include expanded access to multi-month dispensing (MMD), community-based refills, short message service (SMS), and virtual care platforms [3–5].

Significantly, the development of novel long-acting antiretrovirals (LAA), which offer less frequent dosing compared to traditional antiretrovirals (ARVs), represents a breakthrough for HIV care. Injectable cabotegravir and rilpivirine is the first long-acting HIV treatment option to reach high-income markets, with approvals by the United States Food and Drug Administration (US FDA), the European Medicines Agency (EMA), and Health Canada, among others [6–8]. While this product presents challenges for widescale access in LMICs for several reasons, including rilpivirine’s cold chain storage requirements, high virologic failure rates, and monthly or bimonthly clinic visits, research is underway in Kenya, South Africa, and Zimbabwe to investigate the delivery of the injectable regimen in resource-limited settings [9]. More recently, twice-yearly injectable lenacapavir was approved by the US FDA as a treatment option for people living with multi-drug resistant HIV [10]. In addition, there are many other active pharmaceutical ingredients and delivery platforms for both ARVs and broadly neutralizing antibodies being investigated as long-acting treatment options in various clinical and pre-clinical studies, including oral, parenteral, transdermal, and implantable approaches [11].

These new products and innovative service delivery models will be crucial for increasing treatment coverage and retaining PLHIV in care. While significant progress has been made in scaling up ART in LMICs over the past two decades, critical gaps remain. For example, data from the President’s Emergency Plan for Acquired Immunodeficiency Syndrome (AIDS) Relief (PEPFAR) demonstrate that while the total number of people currently receiving ART continues to increase, treatment initiations are decreasing, treatment disruption rates remain high, and an increasing proportion of initiators are non-naïve to ART but are re-entering care [12, 13]. In addition to closing critical treatment and adherence gaps, increased focus on developing and expanding access to new products and interventions also reflects a broader paradigm shift away from one-size-fits-all HIV programs to person-centered care driven by choice, flexibility, and patient preferences. New interventions offer the promise of driving this paradigm shift in the HIV response, accelerating the end of AIDS as a public health threat.

However, this potential will not be realized unless the design, introduction, and rollout of products and services are guided by the voices of community, or end users of care. Moreover, choice also has the potential to increase complexity of HIV treatment programming and may not guarantee benefit to end users if not planned and prioritized appropriately. There are also potential risks involved with expanding choice for HIV treatment, including the potential for more stockouts and supply security concerns with greater fragmentation in the market, greater burden on providers, and higher costs [14–16]. While choice and long-acting products are not likely to enter the HIV treatment landscape for several years, the rollout of long-acting HIV prevention products is slowly beginning in LMICs with the dapivirine vaginal ring and long-acting injectable cabotegravir [17]. Early experiences in HIV prevention will offer valuable lessons to inform planning for HIV treatment, but PLHIV have different perspectives and existing levels of engagement with the health system compared to the end users of HIV prevention services and, therefore, may not have the same preferences for products and service delivery models. As such, it is critical to address the distinct research questions and evidence-generation priorities for PLHIV, apart from those at risk of HIV acquisition.

Conducting targeted research to understand patient acceptability and preferences represents one crucial mechanism for generating evidence on end user experiences. These studies are essential for enabling evidence-based decision-making in program implementation. However, a recent review suggests that the majority of studies that assess patient acceptability in HIV prevention and treatment research lack rigor and do not effectively capture the multi-faceted nature of real-life acceptability [18]. With long-acting products nearing markets, aspects of the HIV response, from supply chains to service delivery, will require a significant redesign. As such, now is a more important time than ever to systematically map the evidence on end user preferences and acceptability to understand what works, what does not, and where there are critical knowledge gaps.

At this pivotal point in the HIV response there is also an urgent need to meaningfully engage communities and advocates further upstream, so they not only provide input on existing interventions, but also inform the broader research agenda based on identified knowledge gaps. A community-led research agenda provides a mechanism for embedding a more nuanced understanding of end user perspectives and experiences into research from the onset, ensuring donors, governments, and other stakeholders can efficiently align future investments with the priorities of communities most affected by HIV.

In this study, we conducted a scoping review to understand the range, characteristics, and coverage of recent HIV treatment preference research, focused on both service delivery attributes and novel ARV formulations. Based on the gaps identified in the literature, we then consulted advocates to develop preliminary recommendations to inform a community-led research agenda. This consultation, held in August 2022, focused on gathering ideas and perspectives on critical evidence generation needs in light of the evolving HIV treatment landscape and paradigm shift towards new treatment modalities and delivery models.

## Methods

### Scoping Review

This review aimed to conduct a rigorous mapping of existing research on the preferences of PLHIV in LMICs for different service delivery models and ART formulations. Ortblad et al.’s review of acceptability assessments in HIV prevention and treatment service delivery research found studies lacked harmonization and rigor [18]. This suggests that a systematic review and meta-analysis may not be suited for this body of literature as empirical evidence cannot be easily collated across diverse methodologies. In a scoping review of key population perspectives on LAAs for treatment and prevention, Sued et al. found that even with this focused population scope, the studies identified were heterogeneous in nature, using variable acceptability measures and methods [19]. Finally, searches through major databases did not identify any reviews focused on HIV treatment product preferences in LMICs. Due to the expected heterogeneity in acceptability literature, the scope of this study covering both products and service delivery attributes, and the lack of previously published reviews on preferences for HIV treatment products among PLHIV in LMICs, we designed this study as a scoping review, complemented by an in-depth analysis on a subset of the literature on product preferences. This scoping review included a systematic literature search and selection of studies, as outlined in the Preferred Reporting Items for Systematic Reviews and Meta-Analyses (PRISMA) guidelines [20–22].

### Literature Search, Eligibility, and Analysis

Because preferences and acceptability are assessed through a wide range of quantitative and qualitative methods, including as sub-objectives in randomized controlled trials, this review took a broad search strategy. The main search was conducted in PUBMED, using key terms for HIV and AIDS, treatment and care, and acceptability and preferences. This was complemented by extracting references from other relevant published reviews. Studies published between January 2014 and May 2022 that reported acceptability or preference data from PLHIV or caregivers for one or more service delivery or product attributes were included in the review. Service delivery attributes are defined broadly, with studies ranging from investigations of preferences for who is providing services to how, when, and where they are being provided. Meanwhile, product attributes refer to any characteristics linked directly to the administration form or specific HIV treatment products.

We took a differentiated approach to eligibility based on whether studies focused on service delivery attributes or products. Across both categories, we included studies that met the following criteria: (1) study populations of PLHIV or caregivers for children living with HIV, and (2) quantitative or qualitative assessment of study population preferences or acceptability for a given intervention or set of intervention options, including both revealed and stated preferences. However, we only restricted eligibility to research focused in LMICs for studies on service delivery attributes. We did not include a geographical limit on studies on treatment products for two reasons. First, initial searches showed that multi-centric trials that included acceptability measures for products did not disaggregate results by country. Second, based on previously published reviews, we hypothesized that literature on product form preferences among PLHIV would be very limited [19].

Studies selected for inclusion were reviewed and details on the objectives, intervention(s) assessed, and acceptability- or preference-related results were extracted. Studies were categorized by intervention focus, methodology, study population, and geography. For studies that included more than one intervention focus (e.g., integrated service delivery and adherence support), we categorized the study under each applicable focus area.

### Consultation and Research Agenda Recommendations

Following completion of the scoping review and preliminary analysis, results were shared during Afrocab’s annual convening in Kigali, Rwanda in August 2022 among 25 PLHIV advocates from 15 countries. With its headquarters in Zambia, Afrocab is a network of community leaders and advocates working to accelerate access to optimal HIV treatments. This session, which included both the presentation of preliminary results and discussion, aimed to inform Afrocab’s workplanning and future advocacy focal areas. Based on results from the literature mapping shared during the presentation, meeting attendees discussed gaps and recommendations on priority areas for future research. Recommendations were summarized and categorized by subject matter. Ethical approval for this activity was not required, as this was conducted as part of a project scoping activity and no data was collected on those involved.

## Results

**Fig. 1:**
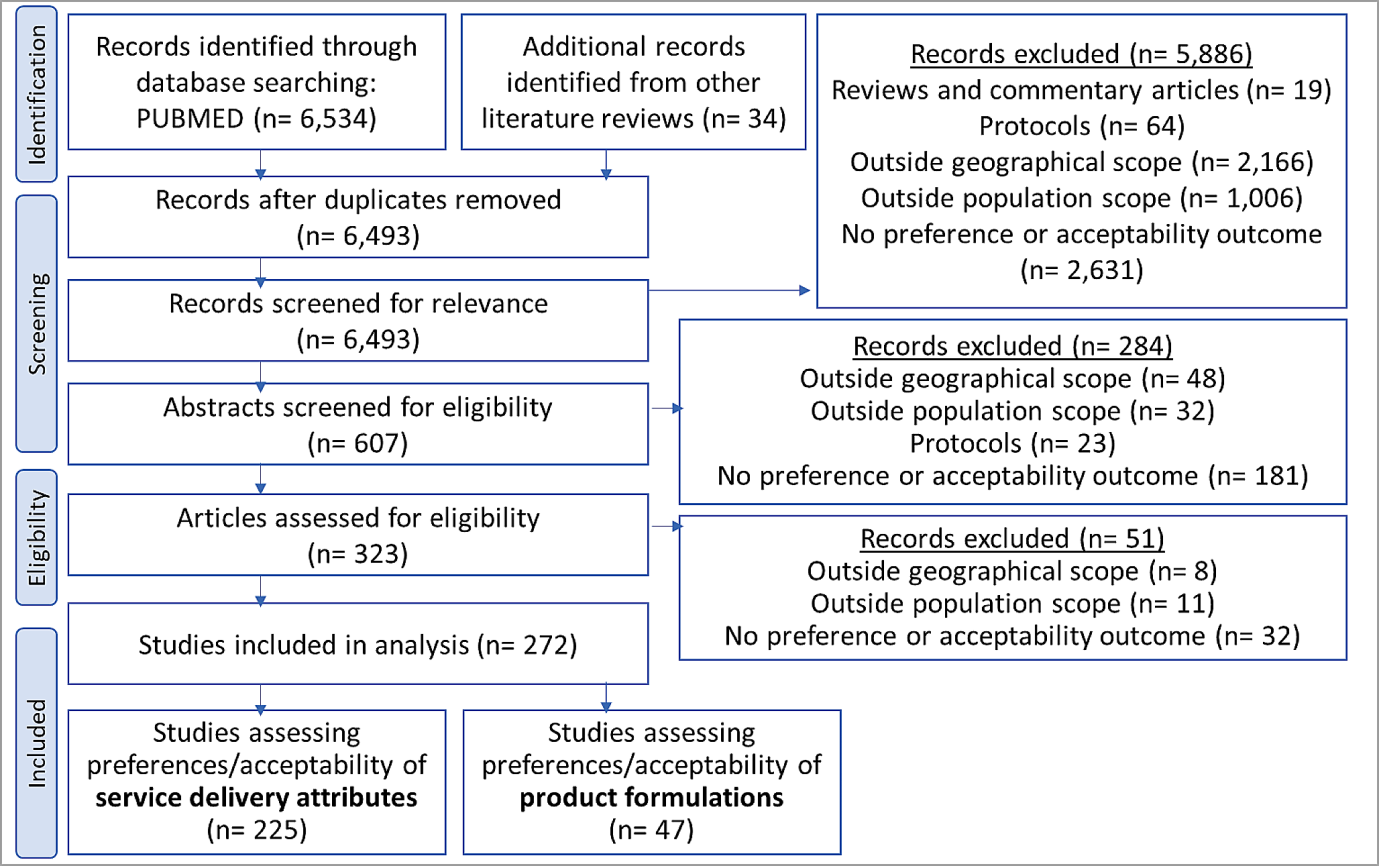
Scoping review screening process. outlines the identification, screening, eligibility, and inclusion criteria for the scoping review

The search yielded 6,493 unique references after screening for duplicates, as shown in Fig. 1. During the screening process, 6,170 records were excluded from the review. The two most prevalent reasons for exclusion were studies conducted among populations outside the review’s geographical focus (e.g., studies on service delivery attributes among PLHIV in high-income countries) and lack of preference or acceptability measure. During the full-text review, 51 additional studies were excluded because they did not include preference or acceptability outcomes, were outside the population of focus (e.g., healthcare providers), or were outside the geographical scope. After an eligibility assessment, 272 studies were included in the review, with 225 studies focused on service delivery attributes and 47 studies on product formulations (see Fig. 1).

**Fig. 2:**
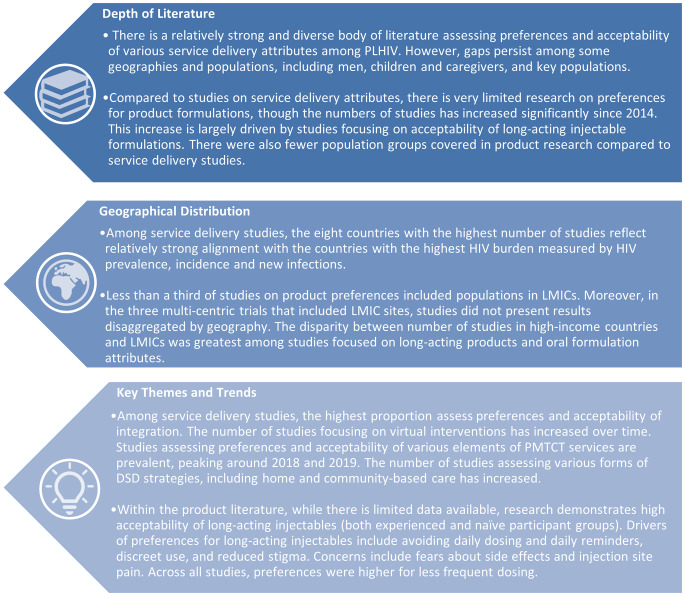
Community-identified areas for further research. summarizes scoping review findings, consolidating insights on depth of literature, geographical distribution, and key themes for service delivery- and product-focused studies

After a decrease from 2014 to 2016, the number of studies published on the preferences among PLHIV steadily increased from 2016 to 2021, with 59% of the studies published since 2019. While this review only included studies published through May 2022, available data from the first half of the year suggest a continuation of this upward trend. Despite wider inclusion criteria (no geographical restrictions), the studies focusing on product preferences were vastly outnumbered by studies on service delivery attributes (in LMICs only). Studies on product preferences only accounted for 17.2% of the literature included in this review, with only five studies published prior to 2018. Key themes across the service delivery-focused studies and product-focused studies are consolidated in Fig. 2.

### Service Delivery-Focused Studies

Characteristics of the studies included are shown in Table 1. Among studies focused on preferences for treatment service delivery attributes, interviews were the most common methodology for eliciting preferences (58.2%, 131/225 studies) followed by surveys (34.6%, 78/225 studies). Over half of studies were published from 2019 onwards, reflecting an increasing trend in preference literature over time.

**Table 1:** Overview of service delivery study characteristics

Categories	Number of studies (*n* = 225)
*Year of Publication*	
2014–2016	58 (25.8%)
2017–2019	78 (34.7%)
2020–2022	89 (39.6%)
*Methodology*	
Quantitative Qualitative	78 (34.7%) 131 (58.2%
Mixed Methods	16 (7.1%)
Study Location**	
South Africa	56 (24.9%)
Kenya	25 (11.1%)
Uganda	19 (8.4%)
Tanzania	15 (6.7%)
Malawi	14 (6.2%)
Nigeria	13 (5.8%)
Zimbabwe	10 (4.4%)
Zambia	9 (4.0%)
All others (30 countries)	71 (31.6%)
Study Population**	
*General Population*	
Adults	98 (43.6%)
Men Only	9 (4.0%)
Women Only	28 (12.4%)
Young People	27 (12.0%)
Children/Caregivers	4 (1.8%)
Pregnant and Breastfeeding People	32 (14.2%)
Key Populations***	
Men Who Have Sex with Men	12 (5.3%)
Transgender People	6 (2.7%)
Sex workers	4 (1.8%)
People Who Use Drugs	9 (4.0%)
Prison Releasees	1 (0.4%)
Study Focus**	
Integration	59 (26.2%)
Technology-based	55 (24.4%)
PMTCT	39 (17.3%)
Adherence support	23 (10.2%)
Peer support	23 (10.2%)
Community groups/delivery	21 (9.3%)
Other DSD	17 (7.6%)

*2022 only included papers published through May 2022, ** In total, more than 100% because some studies collected data in more than one country/location, *** Key populations are defined groups who are at increased risk of HIV, irrespective of the epidemic type or local context

While studies included in the review covered 38 countries in total, they were heavily concentrated geographically. Three-fourths (75.0%) of all studies included in this review focused on populations in Eastern and Southern Africa and nearly a quarter of studies (24.9%) were conducted in South Africa. Nearly all studies (98.2%) focused on a single country context, while four studies (2%) included multiple countries, ranging from two to four countries.

In terms of focal populations, most studies (60.0%) focused on PLHIV over 18 years of age. 27 studies (12.0%) defined their focus as “young people,” but definitions varied across studies, with the most commonly defined study population in this category being 15–24 years (*n* = 5). Few studies (*n* = 4) were among children and/or their caregivers. Among various other priority population groups, the most frequently included populations were pregnant and breastfeeding people (*n* = 32), followed by men who have sex with men (*n* = 12).

Across focal areas, the highest number of studies (26.2%, *n* = 59) assessed preferences for various forms of service integration. Within this category, the most commonly investigated types of integration were mental health screening or care services (*n* = 18), cervical cancer screening (*n* = 14), and sexual and reproductive health services (*n* = 7). Other types of integration included intimate partner violence screening, substance abuse care, non-communicable disease care, and hormone therapy. The second most common focal area involved HIV treatment interventions that leveraged technology-based tools, such as SMS reminders to support adherence and technology-based peer support and counseling mechanisms. The focus on both of these service delivery categories remained relatively consistent over time, making up a high proportion of the studies included in this review throughout the nine-year timespan.

In line with the relatively strong population focus on pregnant and breastfeeding people, the third most frequently assessed study focus was prevention of mother-to-child transmission (PMTCT). The frequency of PMTCT-focused preference studies peaked in 2018–2019, though a wider time frame is needed to validate this trend. Most of the early PMTCT studies assessed acceptability of Option B+ (the initiation of lifelong antiretroviral therapy for all pregnant mothers), while later studies expanded to other areas, particularly technology-based interventions for PMTCT and male involvement [23]. Other areas of care covered in the acceptability literature include adherence support mechanisms, peer support, and community groups or community-based delivery. The focus on various DSD approaches, including community-based delivery models and other mechanisms to optimize service delivery, such as task-shifting, MMD, and youth-friendly services, increased over time.

### Product-Focused Studies

Characteristics of product-focused studies included are shown in Table 2. The number of studies focused on product preferences has increased significantly in recent years, with 66.0% of studies published in 2020 or later. The most frequent methods for preference elicitation included surveys (*n* = 27), interviews (*n* = 15), and discrete choice experiments or other ranking methodologies (*n* = 5).

**Table 2:** Overview of product study characteristics

Categories	Number of studies (*n* = 47)
*Year of Publication*	
2014–2016	5 (10.6%)
2017–2019	11 (23.4%)
2020–2022	31 (66.0%)
*Methodology*	
Quantitative Qualitative	32 (68.1%) 11 (23.5%)
Mixed Methods	4 (8.5%)
Study Location**	
United States	20 (42.6%)
France	9 (19.1%)
Canada	8 (17.0%)
Germany	7 (14.9%)
Spain	7 (14.9%)
South Africa	6 (12.8%)
Italy	6 (12.8%)
Uganda	5 (10.6%)
Netherlands	3 (6.4%)
Australia	3 (6.4%)
United Kingdom	2 (4.3%)
Mexico	2 (4.3%)
Kenya	2 (4.3%)
Argentina	2 (4.3%)
Sweden	2 (4.3%)
Russia	2 (4.3%)
South Korea	2 (4.3%)
Tanzania	1 (2.1%
Dominican Republic	1 (2.1%)
Zimbabwe	1 (2.1%)
Study Population**	
*General Population*	
Adults	35 (74.5%)
Women Only	5 (10.6%)
Young People	3 (6.4%)
Children/Caregivers	6 (12.8%)
*Key Populations*	
Men Who Have Sex with Men	2 (4.3%)
Sex Workers	2 (4.3%)
Study Focus**	
Long-Acting Products	26 (55.3%)
Oral Formulation Attributes	16 (34.0%
Pediatric Formulations	5 (10.6%)

Product-focused studies were heavily concentrated in the US (42.6%, *n* = 20), followed by France (19.1%, *n* = 9), and Canada (17.0%, *n* = 8). Of the 47 studies reviewed, only 15 (31.9%) were conducted among populations in LMICs. This included three multi-centric trials that enrolled participants at sites in LMICs but did not disaggregate results by country. South Africa, Kenya, and Uganda were the only African countries included in more than one study. Ten studies (21.3%) were conducted in multiple countries, ranging from 2 to 13 countries. Most studies focused on adult populations. Unlike service delivery studies, there were no product preference studies among pregnant and breastfeeding women and few among young people, sex workers, and children or caregivers.

### Long-Acting Products

The highest number of studies (*n* = 26) focused on preferences for long-acting products, with most studies investigating patient acceptability for injections. However, among studies on long-acting products, only five included populations in LMICs (two qualitative studies and three multi-centric trials with sites in South Africa and Mexico) [24–28]. Patient-reported outcomes from the three multi-centric trials on cabotegravir and rilpivirine injections, which included patient populations in LMICs, reflect strong preferences for injectable over oral regiments [26–28]. Self-reported outcomes from two studies, ATLAS and FLAIR, show high acceptability of injectable treatment, with statistically significant differences in treatment satisfaction in the injectable arm compared to the oral arm [26, 27]. Moreover, in the exploratory preference question, only 2% of participants in ATLAS and 1% in FLAIR preferred oral treatment to injectable [26]. In week 96 results from ATLAS, satisfaction remained high among those continuing injectable treatment and those who switched from the oral arm [27]. Finally, in patient-reported outcomes from ATLAS-2 M, preferences for injectable over oral treatment were similarly dominant. Comparing dosing options, 94% preferred the bi-monthly injectable, 3% the monthly, and 2% preferred daily oral dosing [28].

The two qualitative studies on preferences for injectable ART similarly demonstrate high interest among both population groups (female sex workers in the Dominican Republic and Tanzania and youth and adults in Coastal Kenya) [24, 25]. Stated preference for long-acting injectable treatment was driven by the desire to avoid pill burden and for easier adherence in both studies. The Kenya study also explored preferences for where injections are delivered, finding that provider-administered injections in clinics were preferred overall, though some youth and MSM expressed interest in home self-administration [25].

### Oral Formulations

12 studies focused on PLHIV’s preferences for various oral formulation attributes, including single versus multiple tablet regimens, side effects, efficacy, and whether the product needs to be taken with food. Only two of these studies included PLHIV in LMICs – Ba et al. (2018) assessed preferences for a single-tablet regimen in a 48-week, open-label trial among adults in Senegal, while Hodes et al. (2018) assessed preferences for various oral formulation attributes among adolescents in South Africa [29, 30]. In the open-label trial in Senegal, acceptability of the single-tablet regimen was found to be high, as evidenced by the high study completion rate [29]. Meanwhile, in the South African study, preferences for various oral pill attributes varied, with most participants preferring white, odorless pills, with discreet packaging [30].

Finally, four studies focused on acceptability of dolutegravir- (DTG-) based regimens. Three studies that measured acceptability of DTG-based regimens were conducted in LMICs – two in Uganda and one in South Africa and Uganda: Nabitaka et al. (2020) reported on patient acceptability of DTG as part of a pilot study in Uganda, Twimukye et al. (2021) conducted in-depth interviews with adults who switched to DTG in Uganda, and Alhassan et al. (2020) conducted in-depth interviews and focus group discussions in South Africa and Uganda among both DTG-experienced and naïve women (and male partners) [31–33]. Across all three studies, DTG was preferred among most participants, in line with the wide acceptance of DTG seen since these studies have been completed. However, most participants reported needing additional support from their providers before and after switching to DTG and feeling rushed to switch to the new regimen [33].

### Pediatric Formulations

Five studies, all of which focused on populations in LMICs, assessed preferences for different pediatric formulations, including minitab sprinkle formulations, syrups, tablets, pellets, and granules [34–38]. Preferences varied across age groups and studies, suggesting that among these options, there was no clear optimal product based on the attributes assessed. Overall, qualitative outcomes showed that, with appropriate training, demonstrations, and guidance, caregivers could rapidly learn to administer new product forms effectively.

### Consultation and Research Agenda Recommendations

During consultation, discussion among advocates affirmed the importance of the growing body of literature on preferences for service delivery models. Advocates highlighted that while there is variation across country contexts, available evidence on the acceptability of innovative models, including MMD and peer- and community-led approaches, has supported important policy changes and driven improved service quality and user satisfaction. However, with long-acting products nearing markets, meeting participants underscored the importance of rapidly filling priority evidence gaps around preferences for these novel technologies and how they are delivered.

**Fig. 3:**
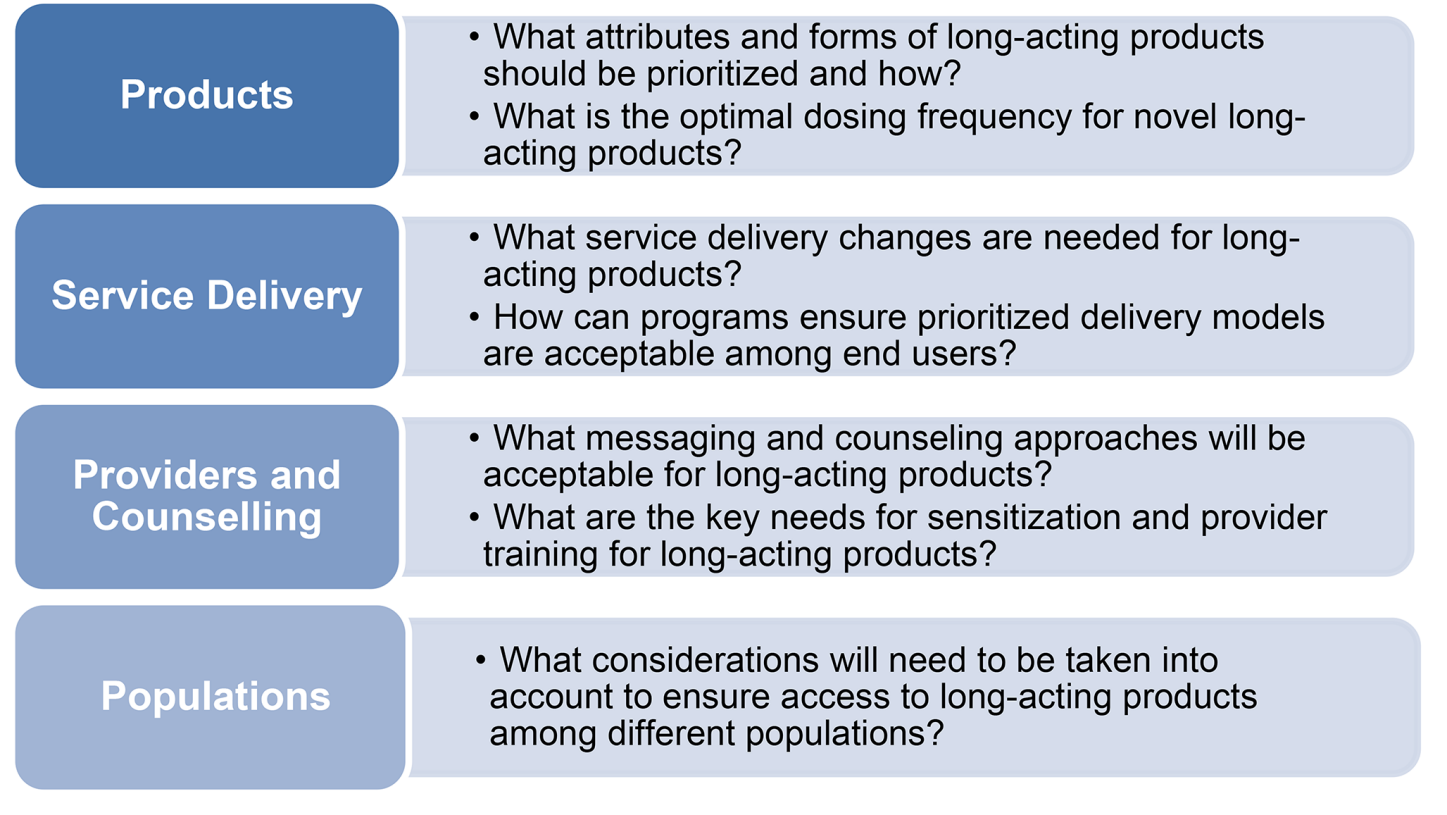
Community-identified areas for further research. depicts the four key areas identified in need of further research during the community consultation and key considerations for discussion amongst each

Advocates identified four key areas where further research is needed: products, service delivery, providers and counseling, and populations (see Fig. 3). For products, meeting participants highlighted that there will be no “one-size fits all option” and more research is needed to ensure programs are equipped to support informed choice in a manner that is acceptable to end users. In terms of product attributes for further investigation, advocates noted that there is a need to generate evidence on acceptability and preferences based on side effect profiles and whether products can be self-administered. For service delivery, discussion focused on the need for additional research on preferences for clinic visit frequency, community-delivery models, and appointment reminders, especially if long-acting products require substantially different follow-up schedules than current regimens. With respect to providers and counseling, recommendations for future research included evidence generation on new communication and counseling tools that address concerns about long-acting products, especially side effects. Finally, discussion among advocates highlighted the importance of understanding preferences for long-acting products among populations that are often excluded from research and early rollout, including pediatric and adolescent populations, pregnant women, and people on second and third-line regimens.

## Discussion

Understanding the preferences of PLHIV for service delivery and product attributes is essential for supporting person-centered care and improving health outcomes. Without sufficient evidence on preferences and acceptability, programs will not be able to efficiently target resources towards the most impactful approaches and interventions. In recent years, HIV programs have made important progress in better aligning services with the preferences and priorities of PLHIV through the expansion of innovative DSD models, though this has taken a slow trajectory. However, the HIV treatment landscape is rapidly changing and the introduction of novel LAAs will require significant adaptations to the health system. Rigorously mapping the available literature on preferences and acceptability for HIV care and consulting the communities most impacted by HIV will help identify the gaps that must be filled to ensure the future landscape of HIV treatment is guided by the priorities of PLHIV.

In this scoping review, we assessed the nature, volume, and characteristics of studies assessing the preferences of PLHIV. Our review found that available literature on the preferences of PLHIV has grown substantially over the past few years, reflecting an increased awareness that HIV programs must be designed to align with the priorities of end users in order to maximize epidemic impact. Our mapping demonstrates that service delivery studies capture end user preferences across a wide range of intervention types and focal areas. However, other reviews focused on assessing methodologies have found that the HIV preference literature lacks rigor and consistency in approach [18, 39]. While the diverse range of service delivery attributes documented in this scoping review may help explain the lack of harmonization, as this body of literature continues to expand, aligning on consistent definitions and approaches for measuring preferences and acceptability will be essential for efficiently comparing results.

Despite the recent regulatory approvals of several long-acting treatment formulations and a growing pipeline of products in development, this review found that the literature on the preferences of PLHIV in LMICs for long-acting products remains extremely limited. Consultation with HIV advocates demonstrated excitement for long-acting products and highlighted that evidence generation on preferences for these novel regimens and how they are delivered should be a focus of future research agendas. However, while evidence generation on preferences for long-acting regimens will be essential for successful new product introduction, there remain critical access barriers that must be addressed to unlock their full potential. Novel long-acting products are currently prohibitively expensive for use in LMICs and market-shaping interventions to increase affordability and accelerate generic development will be critical to ensuring widescale access. Without such interventions, any preference research will remain theoretical and cannot be linked to impact at scale.

There are several important limitations to this study. First, because this review sought to develop a mapping of the availability of evidence on preferences for service delivery and product attributes, there was limited focus on analyzing findings across studies and comparing results, particularly for service delivery studies. Further research that focuses on a narrower scope of studies is needed to better understand the nuances of how preferences differ within intervention types. For example, a review focused solely on preferences for peer-led delivery models can help identify similarities and differences across geographies and populations to inform how existing models can be adapted to suit new contexts and products. Second, initial consultation with advocates aimed to provide high-level recommendations on areas for future research. Further, more intensive consultation is needed to inform the design and execution of research activities. When co-designing new research studies and interventions, consultation should also engage a wider set of stakeholders beyond advocates, including various types of community opinion leaders.

## Conclusions

While preference literature has grown in recent years, particularly for novel service delivery models and interventions, there remain critical gaps around the preferences of PLHIV in LMICs for LAAs and how they are delivered. This scoping review provides a mapping of available literature and evidence gaps, while the consultation provides preliminary recommendations for a future research agenda. Conducting additional research on the preferences of PLHIV, with a research agenda guided by the priorities of advocates and communities, is crucial, alongside critical market-shaping interventions to make LAAs accessible in LMICs.

## Declarations

### Abbreviations

ART

COVID-19

DSD

DTG

LAA

LMIC

MMD

PLHIV

WHO

## References

[1] World Health Organization. Updated recommendations on HIV prevention, infant diagnosis, antiretroviral initiation and monitoring. [Internet]. Geneva: World Health Organization, 2021 [cited 2023 March 2]. https://www.who.int/news/item/17-03-2021-who-publishes-new-clinical-recommendations-on-hiv-prevention-infant-diagnosis-antiretroviral-therapy-initiation-and-monitoring. 33822559

[2] IAS. Person-centered care stakeholder consultation meeting series, 2022–2023. [Internet]. IAS. 2023. https://www.iasociety.org/sites/default/files/PCC/PCC_Consultation_Summary_Report_AIDS_2022_final.pdf.

[3] Boyd AT, Jahun I, Dirlikov E, et al. Expanding access to HIV services during the COVID-19 pandemic—Nigeria, 2020. AIDS Res Ther. 2021;18:62. 10.1186/s12981-021-00385-5. 34538268 10.1186/s12981-021-00385-5 PMC8449993

[4] Holtzman CW, Godfrey C, Ismail L, et al. PEPFAR’s role in protecting and leveraging HIV services in the COVID-19 response in Africa. Curr HIV/AIDS Rep. 2022;19:26–36. 10.1007/s11904-021-00587-6. 34982406 10.1007/s11904-021-00587-6 PMC8724594

[5] Vrazo AC, Golin R, Fernando NB, Killam WP, Sharifi S, Phelps, et al. Adapting HIV services for pregnant and breastfeeding women, infants, children, adolescents and families in resource-constrained settings during the COVID-19 pandemic. J Int AIDS Soc. 2020;23(9):e25622. 32996705 10.1002/jia2.25622 PMC7525801

[6] Johnson and Johnson. Janssen Announces Health Canada Approval of CABENUVA™, the First Long-Acting Regimen for the Treatment of HIV. [Internet]. Johnson and Johnson. https://www.jnj.com/janssen-announces-health-canada-approval-of-cabenuva-the-first-long-acting-regimen-for-the-treatment-of-hiv#:~:text=Cork%2C%20Ireland%2C%20March%2020%2C,HIV%2D1%20infection%20in%20adults.

[7] US FDA. FDA Approves Cabenuva and Vocabria for the Treatment of HIV-1 Infection. https://www.fda.gov/drugs/human-immunodeficiency-virus-hiv/fda-approves-cabenuva-and-vocabria-treatment-hiv-1-infection.

[8] EMA. First long-acting injectable antiretroviral therapy for HIV recommended for approval. [Internet]. EMA. 2020. https://www.ema.europa.eu/en/news/first-long-acting-injectable-antiretroviral-therapy-hiv-recommended-approval.

[9] Matama C. CARES, study newsletter A. 2022. [Internet]. https://jcrc.org.ug/jcrc-bulletin-2021-3-2-2/.

[10] Gilead, Sunlenca^®^. (lenacapavir) Receives FDA Approval as a First-in-Class, Twice-Yearly Treatment Option for People Living With Multi-Drug Resistant HIV. [Internet]. Gilead. 2022. https://www.gilead.com/news-and-press/press-room/press-releases/2022/12/sunlenca-lenacapavir-receives-fda-approval-as-a-firstinclass-twiceyearly-treatment-option-for-people-living-with-multidrug-resistant-hiv#:~:text=(Nasdaq%3A%20GILD)%20today%20announced,MDR)%20HIV%2D1%20infection.

[11] Chandiwana NC, Serenata CM, Owen A, Rannard S, Pérez Casas C, Scott C et al. Impact of long-acting therapies on the global HIV epidemic. AIDS 35(Supplement 2):p S137–43. 10.1097/QAD.0000000000003102 34848580

[12] PEPFAR. PEPFAR Panorama Spotlight. [Internet]. https://data.pepfar.gov/library.

[13] Ehrenkranz P, Rosen S, Boulle A, Eaton JW, Ford N, Fox MP, Grimsrud A, Rice BD, Sikazwe I, Holmes CB. The revolving door of HIV care: revising the service delivery cascade to achieve the UNAIDS 95-95-95 goals. PLoS Med. 2021;18(5):e1003651. 10.1371/journal.pmed.1003651. PMID: 34029346; PMCID: PMC8186775. 34029346 10.1371/journal.pmed.1003651 PMC8186775

[14] Muhoza P, Koffi AK, Anglewicz P, Gichangi P, Guiella G, OlaOlorun F, Omoluabi E, Sodani PR, Thiongo M, Akilimali P, Tsui A, Radloff S. Modern contraceptive availability and stockouts: a multi-country analysis of trends in supply and consumption. Health Policy Plan. 2021;36(3):273–87. 10.1093/heapol/czaa197. PMID: 33454786; PMCID: PMC8058948. 33454786 10.1093/heapol/czaa197 PMC8058948

[15] Politi MC, Estlund A, Milne A, et al. Barriers and facilitators to implementing a patient-centered model of contraceptive provision in community health centers. Contracept Reprod Med. 2016;1:21. 10.1186/s40834-016-0032-3. 29201410 10.1186/s40834-016-0032-3 PMC5693580

[16] Keddem S, Cronholm PD. Peter MD, MSCE, CAQHPMb,c,d. Barriers and Facilitators to Long-Acting Injectable HIV Pre-Exposure Prophylaxis Implementation in Primary Care Since Its Approval in the United States. JAIDS Journal of Acquired Immune Deficiency Syndromes 95(4):p 370–376, April 1, 2024. 10.1097/QAI.0000000000003370. 10.1097/QAI.0000000000003370 PMC10932839 38133586

[17] Jenkins SY, Resar D, Panos Z, Staple A, Watkins M, Ripin D, Amole C. Securing accelerated access to long-acting injectable cabotegravir for HIV prevention in low- and middle-income countries. J Int AIDS Soc. 2023;26(Suppl 2):e26101. 10.1002/jia2.26101. PMID: 37439082; PMCID: PMC10338995. 37439082 10.1002/jia2.26101 PMC10338995

[18] Ortblad KF, Sekhon M, Wang L, Roth S, van der Straten A, Simoni JM, Velloza J. Acceptability Assessment in HIV Prevention and Treatment Intervention and Service Delivery Research: a systematic review and qualitative analysis. AIDS Behav. 2023;27(2):600–17. 35870025 10.1007/s10461-022-03796-1 PMC9868192

[19] Sued O, Nardi N, Spadaccini L. Key population perceptions and opinions about long-acting antiretrovirals for prevention and treatment: a scoping review. Curr Opin HIV AIDS 17(3):p 145–61. 10.1097/COH.0000000000000734 35439789

[20] Moher D, Liberati A, Tetzlaff J, Altman DG, Group P. Preferred reporting items for systematic reviews and meta-analyses: the PRISMA statement. BMJ. 2009;339:b2535. 19622551 10.1136/bmj.b2535 PMC2714657

[21] Arksey H, O’Malley L. Scoping studies: towards a methodological framework. Int J Soc Res Methodol. 2005;8:19–32.

[22] Peters MD, Godfrey CM, Khalil H, McInerney P, Parker D, Soares CB. Guidance for conducting systematic scoping reviews. Int J Evid Based Healthc. 2015;13(3):141–6. 26134548 10.1097/XEB.0000000000000050

[23] Goodluck WL, Mwashemele SZ, Urrio R, Naburi H, Kashmir N, Machumi L, et al. Long-term virological outcomes in women who started option B + care during pregnancy for prevention of mother-to-child transmission of HIV in Dar Es Salaam, Tanzania: a cohort study. Lancet HIV. 2021;8(5):e256–65. 33581776 10.1016/S2352-3018(20)30308-8

[24] Kerrigan D, Sanchez Karver T, Muraleetharan O, Savage V, Mbwambo J, et al. A dream come true: perspectives on long-acting injectable antiretroviral therapy among female sex workers living with HIV from the Dominican Republic and Tanzania. PLoS ONE. 2020;15(6):e0234666. 10.1371/journal.pone.0234666. 32530939 10.1371/journal.pone.0234666 PMC7292359

[25] Simoni JM, Beima-Sofie K, Wanje G, et al. Lighten this Burden of ours: acceptability and preferences regarding injectable antiretroviral treatment among adults and Youth Living with HIV in Coastal Kenya. J Int Assoc Provid AIDS Care. 2021;20:23. 10.1177/23259582211000517 PMC7952847 33685272

[26] Murray M, Antela A, Mills A, Huang J, Jäger H, Bernal E, et al. Patient-reported outcomes in ATLAS and FLAIR participants on long-acting regimens of Cabotegravir and Rilpivirine over 48 weeks. AIDS Behav. 2020;24(12):3533–44. 32447500 10.1007/s10461-020-02929-8 PMC7667137

[27] Swindells S, Lutz T, Van Zyl L, Porteiro N, Stoll M, Mitha E, et al. Week 96 extension results of a phase 3 study evaluating long-acting cabotegravir with rilpivirine for HIV-1 treatment. AIDS. 2022;36(2):185–94. 34261093 10.1097/QAD.0000000000003025 PMC8711605

[28] Chounta V, Overton ET, Mills A, Swindells S, Benn PD, Vanveggel S, et al. Patient-reported outcomes through 1 year of an HIV-1 clinical trial evaluating long-acting cabotegravir and Rilpivirine Administered Every 4 or 8 weeks (ATLAS-2 M). Patient. 2021;14(6):849–62. 34056699 10.1007/s40271-021-00524-0 PMC8563641

[29] Ba S, Raugi DN, Smith RA, Sall F, Faye K, Hawes SE, et al. A trial of a single-tablet regimen of Elvitegravir, Cobicistat, Emtricitabine, and Tenofovir Disoproxil Fumarate for the Initial Treatment of Human Immunodeficiency Virus Type 2 infection in a resource-limited setting: 48-Week results from Senegal, West Africa. Clin Infect Dis. 2018;67(10):1588–94. 29672676 10.1093/cid/ciy324 PMC6927863

[30] Hodes R, Vale B, Toska E, Cluver L, Dowse R. Ashorn, N. Yummy or Crummy?’ The multisensory components of medicines-taking among HIV-positive youth. Glob Public Health. 2019;14(2):284–99. 30067457 10.1080/17441692.2018.1504103

[31] Nabitaka VM, Nawaggi P, Campbell J, Conroy J, Harwell J, et al. High acceptability and viral suppression of patients on Dolutegravir-based first-line regimens in pilot sites in Uganda: a mixed-methods prospective cohort study. PLoS ONE. 2020;15(5):e0232419. 32459822 10.1371/journal.pone.0232419 PMC7252626

[32] Alhassan Y, Twimukye A, Malaba T, Orrell C, Myer L, Waitt C, et al. Community acceptability of dolutegravir-based HIV treatment in women: a qualitative study in South Africa and Uganda. BMC Public Health. 2020;20(1):1883. 33287795 10.1186/s12889-020-09991-w PMC7720619

[33] Twimukye A, Laker M, Odongpiny EAL, et al. Patient experiences of switching from Efavirenz- to Dolutegravir-based antiretroviral therapy: a qualitative study in Uganda. BMC Infect Dis. 2021;21:1154. 34774018 10.1186/s12879-021-06851-9 PMC8590364

[34] Musiime V, Fillekes Q, Kekitiinwa A, Kendall L, Keishanyu R, Namuddu R, et al. The pharmacokinetics and acceptability of Lopinavir/Ritonavir Minitab Sprinkles, tablets, and syrups in African HIV-Infected Children. JAIDS J Acquir Immune Defic Syndr. 2014;66(2):148–54. 24828266 10.1097/QAI.0000000000000135

[35] Kekitiinwa A, Musiime V, Thomason MJ, Mirembe G, Lallemant M, Nakalanzi S, et al. Acceptability of lopinavir/r pellets (minitabs), tablets and syrups in HIV-infected children. Antivir Ther. 2016;21(7):579–85. 27128199 10.3851/IMP3054 PMC6029664

[36] Pasipanodya B, Kuwengwa R, Prust ML, Stewart B, Chakanyuka C, Murimwa T, et al. Assessing the adoption of lopinavir/ritonavir oral pellets for HIV-positive children in Zimbabwe. J Int AIDS Soc. 2018;21(12):e25214. 30549217 10.1002/jia2.25214 PMC6293134

[37] Nebot Giralt A, Nöstlinger C, Lee J, et al. Understanding acceptance of and adherence to a new formulation of paediatric antiretroviral treatment in the form of pellets (LPV/r)-A realist evaluation. PLoS ONE. 2019;14(8):e0220408. 31433803 10.1371/journal.pone.0220408 PMC6703671

[38] Archary M, Zanoni B, Lallemant M, Suwannaprom P, Clarke D, Penazzato M. Acceptability and feasibility of using Raltegravir oral granules for the treatment of neonates in a low-resource setting. Pediatr Infect Dis J. 2020;39(1):57–60. 31815839 10.1097/INF.0000000000002539 PMC11697536

[39] Eshun-Wilson I, Kim HY, Schwartz S, Conte M, Glidden DV, Geng EH. Exploring relative preferences for HIV Service features using Discrete Choice experiments: a synthetic review. Curr HIV/AIDS Rep. 2020;17(5):467–77. 32860150 10.1007/s11904-020-00520-3 PMC7497362

